# Unexplored Bioactive, Antioxidant, Antimicrobial, and Nematicidal Potential of the Flowers, Leaves, and Roots of ‘Yellow Chicory’ (*Hypochaeris sessiliflora*)

**DOI:** 10.3390/ph19081314

**Published:** 2026-08-20

**Authors:** Elena Coyago-Cruz, Gabriela Méndez, Johana Zúñiga-Miranda, Alejandro Porras, Carlos Barba-Ostria, Ivanna Sarabia, Linda P. Guamán, Celina Coello, Jorge Heredia-Moya, Blanca Naranjo, María Claudia Segovia-Salcedo

**Affiliations:** 1 Carrera de Ingeniería en Biotecnología, Universidad Politécnica Salesiana, Sede Quito, Campus El Girón, Av. 12 de Octubre N2422 y Wilson, Quito 170143, Ecuador; 2 Centro de Investigación Biomédica (CENBIO), Facultad de Ciencias de la Salud Eugenio Espejo, Universidad UTE, Quito 170527, Ecuador; 3 Departamento de Ciencias de la Vida y la Agricultura, Universidad de las Fuerzas Armadas ESPE, Quito 171103, Ecuador; 4 Escuela de Medicina, Colegio de Ciencias de la Salud Quito, Universidad San Francisco de Quito (USFQ), Quito 170901, Ecuador; 5 Instituto de Microbiología, Universidad San Francisco de Quito (USFQ), Quito 170901, Ecuador

**Keywords:** Andean region, *Caenorhabditis elegans*, *Candida*, microextraction

## Abstract

This exploratory study compared the flowers, leaves, and roots of *Hypochaeris sessiliflora* to evaluate physicochemical properties, bioactive compounds, in vitro chemical antioxidant activity, antimicrobial activity, and locomotion-disrupting effects on *Caenorhabditis elegans*. Bioactive compounds were determined by liquid chromatography; in vitro chemical antioxidant activity by microplate spectrophotometry; antimicrobial activity against a range of ATCC and multidrug-resistant microorganisms; and locomotion-disrupting effects on *Caenorhabditis elegans*. The results revealed significant differences between plant organs. The leaves contained the highest concentrations of calcium (1163.1 mg/100 g DW), potassium (3256.6 mg/100 g DW), vitamin C (39.2 mg/100 g DW), organic acids (1213.5 mg/100 g DW), and total quantified chlorophylls (89.0 mg/100 g DW). The flowers had the highest levels of total quantified carotenoids (152.1 mg/100 g DM) and total quantified phenolic compounds (12,341.1 mg/100 g DM). They exhibited the highest in vitro chemical antioxidant activity, as measured by the ABTS assay (6.1 mmol TE/100 g DW). The extracts exhibited antimicrobial activity that depended on the plant organ and the microorganism tested, with the root extract being the most active against *Staphylococcus epidermidis* (MIC = 8.2 mg/mL). No activity was detected against *Candida* species or against multidrug-resistant bacteria under the experimental conditions used. Furthermore, the extracts under the experimental conditions used did not significantly alter *C. elegans’* locomotion. Overall, this study provides baseline evidence of the organ-specific chemical composition and biological properties of *H. sessiliflora*, supporting further investigation of the compounds responsible for the observed in vitro chemical antioxidant and selective and antimicrobial activities.

## 1. Introduction

Plants constitute one of the most important reservoirs of structurally diverse natural products, many of which cannot be easily reproduced by conventional synthetic chemistry. Over the past decades, phytochemicals such as phenolic acids, flavonoids, terpenoids, and alkaloids have provided key scaffolds for clinically used drugs and have inspired the development of novel therapeutic agents in oncology, infectious diseases, and inflammatory disorders [1,2]. In this context, the systematic study of underutilised and endemic plant species not only broadens the portfolio of bioactive molecules available for food, pharmaceutical, and biotechnological applications but also contributes to the conservation and valorisation of local biodiversity, in line with global strategies for a sustainable bioeconomy.

Within this framework, the *Asteraceae* family, one of the largest families of flowering plants, includes numerous species widely used as food and in traditional medicine, such as chicory, sunflower, lettuce, and chamomile [3]. These plants are recognised as important sources of vitamins, phenolic compounds, carotenoids, chlorophylls, and other secondary metabolites associated with antioxidant, antimicrobial, and anti-inflammatory properties [4]. Although several *Asteraceae* species have been characterised from a nutritional and functional perspective, many high-Andean taxa remain poorly studied despite their integration into local culinary practices and folk remedies. The extreme environmental conditions of high-altitude ecosystems, including intense solar radiation, low temperatures, and marked daily thermal fluctuations, may favour the accumulation of protective metabolites, making these native species promising sources of health-promoting compounds [5].

Within the family of Asteraceae, the genus *Hypochaeris* comprises approximately 100 species, most of which are native to South America. Several species of this genus are of culinary and medicinal interest. For example, *Hypochaeris laevigata* var. *hipponensis* has a bitter-tasting root that is eaten in salads. Meanwhile, *Hypochaeris radicata* is characterised by its wide variety of secondary metabolites, including alkaloids, flavonoids, glycosides, cardiac glycosides, phenolic compounds, resins, saponins, steroids, tannins, terpenoids and triterpenes. These compounds have been linked to various biological activities, including anti-inflammatory, anti-cancer, antioxidant, antibacterial, and antifungal effects. Furthermore, this species is used in traditional medicine to treat conditions such as jaundice, rheumatism, dyspepsia, constipation, hypoglycaemia and kidney disorders [6].

In this context, *Hypochaeris sessiliflora* Kunth, commonly known as ‘yellow chicory’, ‘achicoria’, ‘chikku chikku’ and ‘sañi’, is a native high-Andean species of the Asteraceae family distributed along the Andes from Venezuela to Chile, where it grows at altitudes ranging from approximately 1430 to 5100 m above sea level [7]. In rural Andean communities, species of the genus *Hypochaeris* are consumed as vegetables or as infusions and used in traditional medicine; however, scientific information on the chemical composition and bioactive potential of *H. sessiliflora* remains limited [8]. This knowledge gap is particularly relevant in the current context of increasing interest in plant-derived bioactive compounds as natural antioxidants and antimicrobial agents, especially given the global rise in antimicrobial resistance associated with the extensive use and misuse of antibiotics in human and veterinary medicine [9].

Whilst the phytochemical and antioxidant properties of related species, such as *H. radicata* and *H. laevigata*, have been investigated, these findings cannot be directly extrapolated to *H. sessiliflora*. There is still limited evidence integrating its physicochemical characteristics, mineral profile and specific classes of bioactive molecules with functional assays such as antioxidant capacity, antimicrobial activity against reference and multidrug-resistant bacteria, and activity that alters nematode locomotion. Obtaining these data is necessary to establish an initial chemical and functional profile of ‘yellow chicory’ and to determine whether its different plant organs warrant further investigation as potential sources of bioactive compounds.

Therefore, this exploratory study aimed to comparatively characterise the flowers, leaves and roots of *H. sessiliflora* by determining their physicochemical properties, mineral composition, major bioactive compounds, in vitro chemical antioxidant capacity using complementary radical scavenging methods, antimicrobial assays against reference bacterial strains to determine the initial inhibitory spectrum, assays against *Candida* species and multidrug-resistant bacterial isolates, and detectable alterations in the locomotion of the nematode *Caenorhabditis elegans*. The study thus established a specific initial chemical and functional profile for each organ of *H. sessiliflora*, which could be prioritised in subsequent metabolomic, fractionation and bioactivity-guided research.

## 2. Results

### 2.1. Chemical Analysis of Hypochaeris sessiliflora

Table 1 presents the preliminary phytochemical screening of the aqueous extract prepared from the flowers, leaves, and roots of *H. sessiliflora*. Preliminary qualitative phytochemical screening produced a presumptive positive response for steroids only in the flower aqueous extract. Presumptive positive responses for phenolic compounds and tannins were observed in the flower and root extracts. In contrast, saponins produced positive froth-test responses in the extracts of all three plant organs. No characteristic reaction was observed for the remaining metabolite classes under the screening conditions used.

Table 2 presents the average values from chemical analysis and mineral concentrations in the flowers, leaves, and roots of *H. sessiliflora*. The results showed that the three parts assessed had average pH values of 5.8 and titratable acidity of 0.1%, with no statistically significant differences between them. The roots had the highest concentration of soluble solids, at 2.7 °Brix. In terms of moisture content, the flowers and leaves had the highest values, with an average of 82.9%, whilst the roots showed a lower content (69.3%). Regarding ash content, the leaves had the highest concentration (2.3%), followed by the flowers (1.2%). Furthermore, no quantifiable concentrations of iron were detected in the leaves and roots. On the other hand, the leaves had the highest concentrations of calcium (1163.1 mg/100 g DW) and potassium (3256.6 mg/100 g DW).

### 2.2. Bioactive Compounds of Hypochaeris sessiliflora

Table 3 presents the average values of bioactive compounds present in the flowers, leaves, and roots of *H. sessiliflora*. The results showed that the flowers and leaves had the highest concentrations of vitamin C, averaging 37.4 mg/100 g DW, with no statistically significant differences between the two plant parts. About organic acids, the leaves recorded the highest total concentration, followed by the flowers and then the roots. Furthermore, the predominant acids in the leaves were malic and tartaric acids.

The most common carotenoids were α-carotene, lutein, 9-cis-antheraxanthin, zeaxanthin and β-carotene. The highest concentrations of α-carotene and β-carotene were found in the flowers. Meanwhile, chlorophylls and derivatives, including chlorophyll b and pheophytin a, were present at high concentrations in the leaves.

Furthermore, total quantified phenolic compounds were highest in the flowers, followed by the leaves and then the roots. The predominant phenolic compounds identified were gallic acid, 4-hydroxybenzoic acid, *m*-coumaric acid, syringic acid, ferulic acid, rutin and kaempferol. The most predominant compound was ferulic acid, detected in the flowers at a concentration of 11,902.7 mg/100 g DW. The second most abundant phenolic compound in the roots was *m*-coumaric acid, at 531.6 mg/100 g DW, followed by rutin in the leaves, at 435.7 mg/100 g DW.

### 2.3. In Vitro Chemical Antioxidant Activity of Hypochaeris sessiliflora

Table 4 presents the average in vitro chemical antioxidant activity values in the flowers, leaves, and roots of *H. sessiliflora*. The results showed an average in vitro chemical antioxidant activity of 2.9 mmol TE/100 g DW, as determined by the DPPH method, with no statistically significant differences between the three parts assessed. On the other hand, the ABTS method revealed higher in vitro chemical antioxidant activity in the flowers, followed by the leaves and then the roots. Furthermore, when comparing the two methods, the ABTS values were higher than those from the DPPH assay.

### 2.4. Antimicrobial Activity of Hypochaeris sessiliflora

#### 2.4.1. ATCC Microorganisms

Hydroethanolic extraction yields from the flowers, leaves, and roots were 11.1%, 32.5%, and 7.1%, respectively. The leaf material showed the highest total phenolic content on a plant dry-weight basis. However, when expressed per gram of dry extract, the flower extract presented the highest concentration, followed by the leaf and root extracts (Table 5).

Table 6 presents the minimum inhibitory concentrations (MICs) of the flower, leaves, and roots extracts of *H. sessiliflora* against the evaluated bacterial strains. Leaves and roots each inhibited eight of the eleven strains, although their activity profiles differed, whereas the flower extract inhibited five strains. All three extracts inhibited *E. coli*, *S. aureus*, *L. monocytogenes* and *E. tarda*. None of the extracts inhibited *P. fluorescens* or *P. putida* at the highest concentration tested.

Leaf extracts inhibited *E. coli*, *P. aeruginosa*, *S. aureus*, *S. epidermidis*, *E. faecalis*, *K. pneumoniae*, *L. monocytogenes*, and *E. tarda*. The roots inhibited the same number of strains but showed no detectable activity against any of the three *Pseudomonas* species. The flower extracts inhibited *E. coli*, *S. aureus*, *S. mutans*, *L. monocytogenes*, and *E. tarda*. The lowest recorded MIC was 8.2 mg/mL for the root extract against *S. epidermidis*. However, most MIC values were above 30 mg/mL, indicating that relatively high extract concentrations were required to inhibit bacterial growth.

#### 2.4.2. Anticandidal Activity

None of the flower, leaf, or root extracts showed detectable antifungal activity against *Candida albicans*, *C. tropicalis*, *C. krusei*, or *C. glabrata* at any of the tested concentrations.

#### 2.4.3. Multidrug-Resistant Microorganisms

In this study, the flowers, leaves, and roots of *H. sessiliflora* were evaluated against multidrug-resistant strains of *Klebsiella pneumoniae*, *Escherichia coli*, *Salmonella enterica serovar Kentucky*, *Enterococcus faecalis*, *Staphylococcus epidermidis*, *Enterococcus faecium*, and *Pseudomonas aeruginosa* using a broth microdilution assay. No detectable antibacterial activity was observed at the tested concentrations, suggesting that, under these experimental conditions, *H. sessiliflora* does not exert a direct inhibitory effect on the growth of these highly resistant pathogens. However, the concentration range was constrained by the solubility in DMSO of the crude extracts; the maximum concentrations assayed (125 µg/mL for flowers and leaves and 2 mg/mL for roots) corresponded to the highest levels at which homogeneous dispersions could be obtained without precipitation, which may have limited the detection of potential antibacterial effects. Therefore, these negative findings should be interpreted with caution, as they do not rule out the possibility that higher concentrations, alternative solubilization approaches, or fractionation of *H. sessiliflora* extracts could reveal antibacterial activity, particularly against less-resistant strains or under different assay conditions.

### 2.5. Locomotion-Disrupting Effects of Hypochaeris sessiliflora Hydroethanolic Dry Extracts

**Figure 1:**
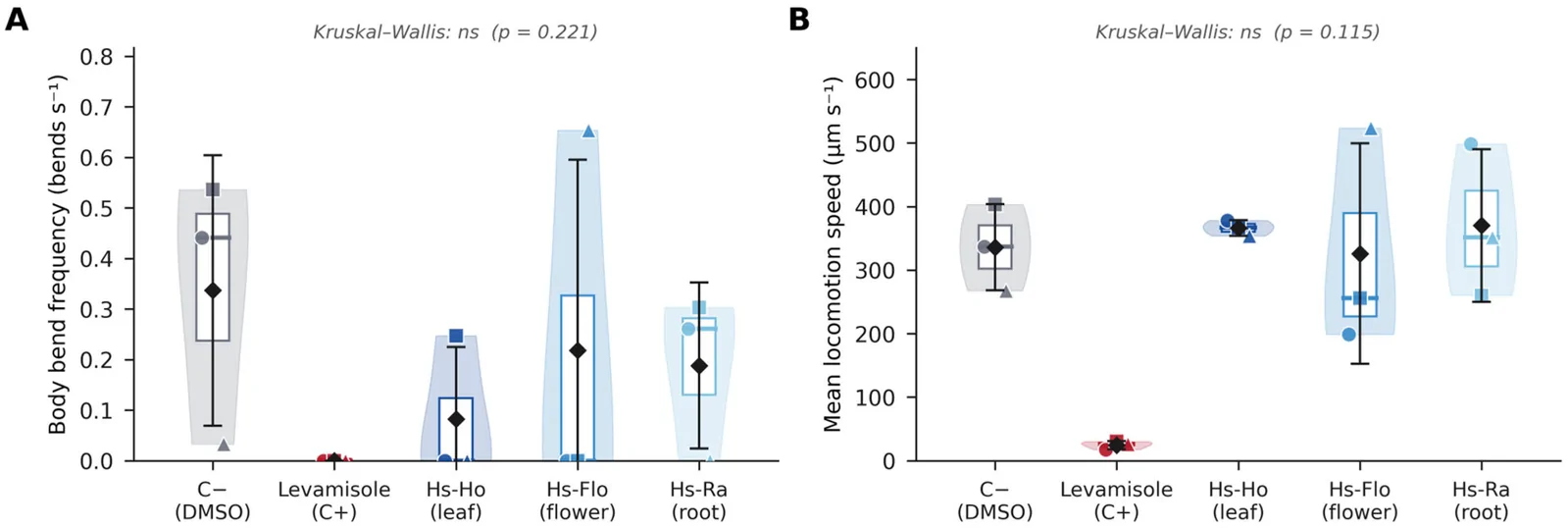
**Effects of *Hypochaeris sessiliflora* hydroethanolic dry extracts on *Caenorhabditis elegans* locomotion.** Body-bend frequency (**A**) and mean locomotion speed (**B**) were measured in wild-type *C. elegans* N2 animals treated with *H. sessiliflora* leaf (Hs-Ho, 500 µg mL^−1^), flower (Hs-Flo, 125 µg mL^−1^), or root (Hs-Ra, 500 µg mL^−1^) hydroethanolic dry extracts. Vehicle-treated animals were used as negative control (C−; 2.5% DMSO), and levamisole as positive control (C+; 20 µg mL^−1^). Three independent 20 s videos were analysed per condition. Each point represents the median value across all tracks detected in a single video and was used as the unit of analysis. Circles, squares, and triangles indicate Videos 1, 2, and 3, respectively. Violin plots show the distribution of per-video medians; white boxes indicate the interquartile range, horizontal lines the median, black diamonds the mean, and error bars ±1 SD. Colours indicate conditions: grey, C−; red, C+; dark blue, Hs-Ho; medium blue, Hs-Flo; light blue, Hs-Ra. No significant differences were detected between any *H. sessiliflora* extract and C− by pairwise Mann–Whitney U tests. Kruskal–Wallis H and *p* values are shown above each panel. Measurements were calibrated using 280 pixels = 1000 µm. BBPS is expressed as bends s^−1^ and AvgSpeed as µm s^−1^.

The effect of hydroethanolic dry extracts prepared from *H. sessiliflora* leaves (Hs-Ho), flowers (Hs-Flo), and roots (Hs-Ra) on *Caenorhabditis elegans* N2 locomotion was assessed from 20 s video recordings. Three independent videos were analysed per condition, and the median value of all tracks detected in each video was used as the unit of analysis. Body-bend frequency (BBPS) and mean locomotion speed (AvgSpeed) are shown in Figure 1A,B. Normalised speed (BLPS) and cumulative track distance are presented in Figure S1A,B.

Under vehicle control conditions (C−; 2.5% DMSO), worms showed median AvgSpeed values of 267.6, 337.0, and 403.2 µm s^−1^, corresponding to a mean ± SD of 335.9 ± 67.8 µm s^−1^. The corresponding BBPS values were 0.033, 0.441, and 0.536 bends s^−1^, with a mean of 0.337 ± 0.267 bends s^−1^. BLPS was 0.125 ± 0.033 body lengths s^−1^, and cumulative track distance was 182.5 ± 39.3 µm. The proportion of active tracks was 94.4 ± 1.8%.

Levamisole-treated animals (C+; 20 µg mL^−1^) showed the expected reduction in locomotion. BBPS was 0.000 ± 0.000 bends s^−1^ in all three videos, whereas AvgSpeed was reduced to 24.6 ± 6.4 µm s^−1^. BLPS and cumulative track distance were 0.015 ± 0.008 body lengths s^−1^ and 5.1 ± 7.0 µm, respectively. The proportion of active tracks was 39.4 ± 29.7%.

Treatment with the leaf extract Hs-Ho did not reduce locomotion relative to the vehicle control. AvgSpeed values were 353.9, 367.0, and 378.3 µm s^−1^, with a mean of 366.4 ± 12.2 µm s^−1^ (Figure 1B). BBPS values were 0.000, 0.000, and 0.247 bends s^−1^, with a mean of 0.082 ± 0.143 bends s^−1^ (Figure 1A). BLPS was 0.133 ± 0.024 body lengths s^−1^, and cumulative track distance was 194.9 ± 40.7 µm (Figure S1A,B). The proportion of active tracks was 94.3 ± 1.6%.

Treatment with the flower extract Hs-Flo also produced no consistent reduction in locomotion. AvgSpeed values were 198.8, 255.7, and 523.6 µm s^−1^, with a mean of 326.0 ± 173.4 µm s^−1^ (Figure 1B). BBPS values were 0.000, 0.000, and 0.654 bends s^−1^, with a mean of 0.218 ± 0.377 bends s^−1^ (Figure 1A). BLPS was 0.128 ± 0.102 body lengths s^−1^, and cumulative track distance was 172.8 ± 53.5 µm (Figure S1A,B). The proportion of active tracks was 95.6 ± 2.6%.

Treatment with the root extract Hs-Ra yielded AvgSpeed values of 260.2, 351.7, and 498.7 µm s^−1^, with a mean of 370.2 ± 120.3 µm s^−1^ (Figure 1B). BBPS values were 0.000, 0.261, and 0.303 bends s^−1^, with a mean of 0.188 ± 0.164 bends s^−1^ (Figure 1A). BLPS was 0.137 ± 0.042 body lengths s^−1^, and cumulative track distance was 172.5 ± 63.9 µm (Figure S1A,B). The proportion of active tracks was 96.5 ± 1.5%.

Kruskal–Wallis analysis across the five conditions showed no significant differences for BBPS (H = 5.716, *p* = 0.221), AvgSpeed (H = 7.433, *p* = 0.115), BLPS (H = 7.233, *p* = 0.124), or cumulative track distance (H = 7.300, *p* = 0.121). Pairwise Mann–Whitney U comparisons between the vehicle control and each *H. sessiliflora* extract yielded uncorrected *p*-values ≥ 0.184 for all parameters. Rank-biserial correlation coefficients ranged from −0.778 to +0.333. In contrast, comparison between the vehicle control and levamisole yielded r = −1.000 for all four locomotion parameters.

Together, these results show that hydroethanolic dry extracts from *H. sessiliflora* leaves, flowers, and roots did not significantly alter adult *C. elegans* locomotion under the conditions evaluated.

## 3. Discussion

### 3.1. Chemical Analysis of Hypochaeris sessiliflora

A phytochemical screening of *Hypochaeris sessiliflora*, known locally as ‘yellow chicory’, provides only preliminary differential distribution of secondary metabolites among flowers, leaves, and roots, reflecting the physiological role of each plant organ. Because the screening was performed using aqueous extracts, metabolites with limited water solubility may have been underrepresented. Thus, flowers generally accumulate metabolites associated with protection against ultraviolet radiation, chemical defence, and pollinator interactions [4,5]. In contrast, roots tend to concentrate compounds involved in defence against soil microorganisms and rhizosphere interactions [10,11].

Phytochemical information on *H. sessiliflora* remains limited. Previous studies on medicinal species native to Ecuador have reported limited chemical characterisation, highlighting the relevance of the present results as an initial contribution to their phytochemical profiles [12,13]. In addition, *H. sessiliflora* belongs to the Asteraceae family, which is widely distributed in high-Andean ecosystems where intense solar radiation, low temperatures, and environmental stress can promote the synthesis of protective secondary metabolites [7,14].

Phenols and tannins detected in flowers and roots suggest that these metabolites may contribute to the antioxidant capacity of these tissues. In Asteraceae, phenolic compounds are among the most abundant and biologically relevant metabolites because of their role in free radical scavenging. Recent studies in this family have reported a broad diversity of phenolic compounds together with a positive association between phenolic content and antioxidant activity [15].

Saponins were detected in flowers, leaves, and roots, making them the most widely distributed metabolites in *H. sessiliflora*. Their presence across all organs suggests that they may form part of a constitutive chemical defence system. Due to their amphipathic nature and ability to interact with biological membranes, saponins have attracted interest for their antimicrobial and antifungal properties. Recent studies on medicinal plant extracts have identified saponins, tannins, and phenolic compounds among the phytochemical groups with the highest biological relevance [16].

Soluble solids differed markedly among plant organs, with roots showing the highest value (2.7 °Brix). This result is likely associated with the storage function of roots and the accumulation of soluble sugars, organic acids, and other water-soluble compounds. In high-Andean plants, the distribution of these compounds is influenced by plant organ, phenological stage, and environmental conditions such as low temperatures, intense solar radiation, and water stress, all of which affect primary and secondary metabolism [12]. Furthermore, species of the genus *Hypochaeris* showed values of 0.6 °Brix, 5.2% and 4.5% titratable acidity, and 6.0 pH in basal rosettes [2], which were low for soluble solids and high for titratable acidity and pH, compared with the values obtained in this study.

Moisture content was significantly higher in flowers and leaves, consistent with the elevated metabolic activity of these tissues. Leaves and flowers are directly involved in photosynthesis, transpiration, growth, and reproduction, processes that require higher water availability. In contrast, roots had lower moisture levels, possibly due to a greater proportion of dry matter, structural components, and reserve metabolites [17].

Ash content was highest in leaves, followed by flowers and roots, indicating greater mineral accumulation in leaf tissues. This pattern agrees with the physiological role of leaves in photosynthesis, osmotic regulation, and ion exchange [18]. The higher ash content also coincided with the highest Ca and K concentrations detected in leaves. In Asteraceae species, calcium and potassium are commonly reported as predominant macronutrients, although their levels vary by species, plant organ, soil characteristics, and environmental mineral availability [19].

### 3.2. Bioactive Compounds of Hypochaeris sessiliflora

Table 3 shows a clear chemical differentiation among flowers, leaves, and roots of *Hypochaeris sessiliflora*. Vitamin C concentration was higher in flowers and leaves, consistent with previous studies reporting elevated ascorbic acid levels in leaves compared with flowers and stems [20].

Regarding organic acids, leaves presented the highest total concentration, followed by flowers and roots. Tartaric and malic acids were the predominant compounds in leaves. Similar findings have been reported in species of the family Asteraceae, in which organic acids and other primary and secondary metabolites have been identified across different plant organs [21].

The carotenoid profile revealed the presence of lutein, 9-cis-antheaxanthin and zeaxanthin in flowers and leaves, whilst only lutein was detected in the roots. This distribution is consistent with the typical localisation of these pigments in photosynthetic tissues or those with a pigmentary function. Carotenoids play an important role in both the colouration of plant tissues and their bioactive potential. In this context, the presence of lutein, violaxanthin and β-carotene has been reported in various floral tissues [22]. Similarly, studies carried out on species of the genus *Hypochaeris* reported total quantified carotenoid contents of 351.8 and 449.5 mg of β-carotene per 100 g of fresh weight (FW) [23].

Chlorophylls and their derivatives were detected exclusively in flowers and leaves, whilst they were not identified in the roots. This pattern is consistent with the photosynthetic function of aerial organs, in which these pigments capture light energy and participate in photosynthesis.

Phenolic compounds represented the most predominant group identified in Table 3. Flowers showed the highest total quantified phenolic concentration, followed by leaves and roots. Ferulic acid was the predominant compound in all three organs, particularly in flowers. In Asteraceae species, phenolic acids and flavonoids, including ferulic acid, *p*-coumaric acid, rutin, and kaempferol, have been described as predominant components of the bioactive profile [15]. Furthermore, studies on *Hypochaeris* species reported total quantified phenolic contents ranging from 946.5 to 1103.6 mg GAE/100 g FW [23].

### 3.3. Antioxidant Activity of Hypochaeris sessiliflora

The antioxidant activity of *H. sessiliflora* varied depending on the analytical method used and the plant part assessed. The differences observed have been widely described in studies of bioactive compounds, as DPPH and ABTS respond differently to antioxidant mechanisms and the chemical nature of the metabolites present. The DPPH radical has a greater affinity for lipophilic compounds. It is often limited in its ability to detect hydrophilic antioxidants or complex mixtures, whereas ABTS can detect both hydrophilic and lipophilic antioxidants [24].

The flower and leaf samples showed higher ABTS values than the roots. The compositional analysis of the corresponding antioxidant assay extracts identified bioactive compunds compounds (Table 3); however, their simultaneous presence does not establish that any individual compound caused the measured radical-scavenging response. The higher ABTS values in flowers and leaves are consistent with the previously observed phytochemical profile, as both tissues exhibited higher concentrations of total quantified phenolic compounds and greater diversity of bioactive metabolites. In particular, the flowers had the highest phenolic content, dominated by ferulic acid, rutin, and kaempferol, whilst the leaves contained higher concentrations of vitamin C, organic acids, ferulic acid, and rutin. Thus, other authors have demonstrated that phenolic acids and flavonoids, such as ferulic acid, rutin, and kaempferol, contribute significantly to the antioxidant capacity as determined by ABTS and DPPH [15]. In turn, the lower ABTS activity observed in the roots also coincides with their lower total quantified phenolic compound concentration. Although the roots contained *m*-coumaric acid and ferulic acid, the total quantified phenolic content was considerably lower than that of the flowers and leaves. This suggests that individual organic acids, carotenoids, chlorophylls and their derivatives, as well as phenolic compounds, may contribute in different ways to the responses measured in antioxidant and antimicrobial assays. Although some grouped variables showed statistical associations with these responses, such associations do not establish that the corresponding chemical class was responsible for the observed activity.

Similar organ-dependent differences have been reported in other Asteraceae species, in which inflorescences and leaves differed in polyphenol content and showed distinct responses to ABTS and DPPH, indicating differences in the polarity and composition of their antioxidant fractions [3].

The antioxidant assessment was limited to the chemical ABTS and DPPH assays. Although these methods allow comparison of the radical-scavenging capacity of the extracts, they do not establish antioxidant effects in biological systems. Future studies should evaluate the extracts in appropriate cell-based oxidative stress models, including measurements of intracellular reactive oxygen species, oxidative damage, and cytotoxicity, to determine their biological relevance and functional antioxidant potential.

### 3.4. Antimicrobial Activity of Hypochaeris sessiliflora

*Hypochaeris sessiliflora* is a perennial herb of the Asteraceae family. Although Asteraceae species are widely recognised as sources of phenolic acids, flavonoids, terpenoids, and other secondary metabolites with reported biological effects [22,25], the biological properties of *H. sessiliflora* remain poorly characterised. Because the assays were performed using crude extracts, the measured responses represent the combined effects of complex chemical mixtures.

The analysis of the hydroethanolic extracts used in the biological assays showed marked organ-dependent differences in total phenolic content. Nevertheless, this variable did not predict the antimicrobial response. Consequently, the contribution of individual compounds and possible additive, synergistic, or antagonistic interactions could not be determined. Identification of the active constituents would require bioactivity-guided fractionation, purification, and evaluation of individual compounds and reconstructed mixtures at concentrations equivalent to those present in the original extracts.

The antibacterial results should be interpreted cautiously. Although several strains were inhibited, most MIC values ranged from 31.2 to 162.5 mg/mL. The lowest values were recorded for the root extract against *S. epidermidis* (8.2 mg/mL), the flower and leaf extracts against *E. tarda* (12.8 and 14.5 mg/mL, respectively), and the leaf extract against *S. aureus* (15.6 mg/mL). Nevertheless, these values still represent relatively high mass concentrations for crude extracts and do not demonstrate pharmacological or therapeutic efficacy. Other Asteraceae extracts evaluated by broth microdilution have shown MIC values of 0.125–4 mg/mL [26]; however, differences in species, extraction procedures, inoculum, culture medium, and endpoint determination prevent direct equivalence. The present findings should therefore be considered preliminary evidence of selective in vitro growth inhibition that may guide subsequent fractionation rather than evidence of antibiotic potential [27].

Hydroethanolic dry extracts from flowers, leaves, and roots of *H. sessiliflora* did not inhibit the growth of *Candida albicans*, *C. tropicalis*, *C. krusei*, or *C. glabrata* at the concentrations tested, from 78.125 to 1250 µg mL^−1^. This result was observed for all three plant parts, despite differences in their phytochemical profiles. Thus, under the assay conditions used here, the extracts showed no detectable anti-*Candida* activity. This result differs from reports on *H. radicata*, a related species whose leaf and root extracts showed antifungal activity against a panel that included *C. albicans* and several filamentous fungi [28]. However, that study used different plant material, extraction schemes, and assay conditions. Therefore, its results cannot be directly extrapolated to *H. sessiliflora*. The negative result obtained here should be interpreted as specific to the hydroethanolic dry extracts, test organisms, and concentration range evaluated. Because antimicrobial activity was evaluated using crude extracts, the observed MIC values represent the net response of a complex mixture. Consequently, the presence or concentration of a single compound in an active extract does not demonstrate that the compound was responsible for bacterial inhibition.

The absence of anti-*Candida* activity should not be inferred directly from the chemical composition of the extracts. Ferulic acid, caffeic acid, and related phenolic acids have been reported to show anti-*Candida* activity when tested as pure compounds [29]. Kaempferol has also been shown to inhibit *C. glabrata* growth, although with lower activity than the reference antifungal used in that study [30]. Nevertheless, the presence of these metabolites in a crude extract does not predict antifungal efficacy. Their final concentrations, chemical forms, and interactions with other constituents may differ from those of purified standards. Although phenolics were detected in the hydroethanolic extract, the tested mixtures did not inhibit *Candida* growth under the evaluated conditions. This result does not establish the activity or inactivity of the individual compounds because they were not tested separately.

Saponins were detected in all three plant parts, but their presence also does not necessarily imply antifungal activity. Studies with defined C-27 steroidal saponins showed that anti-*Candida* activity depends on the aglycone and on the number and structure of sugar residues; several saponins and all tested sapogenins were inactive at the highest concentration evaluated [31]. Since the saponins of *H. sessiliflora* were not structurally characterised, it is not possible to assign them to active or inactive subclasses. The lack of activity in the leaf extract, in which only the secondary metabolite class of saponins was detected, supports the need for structural and functional characterisation before assigning a biological role to this fraction.

Taken together, these data define a clear boundary for the antimicrobial profile of *H. sessiliflora*: the hydroethanolic dry extracts tested here were inactive against the four *Candida* species examined. This does not exclude activity in other fractions, at higher concentrations, or against other fungal targets. It does show that the antibacterial activity observed elsewhere in this study does not extend to *Candida* under the conditions evaluated. Further work should therefore focus on bioassay-guided fractionation only if antifungal activity is recovered in alternative extracts or enriched fractions.

### 3.5. Locomotion-Disrupting Effects of Hypochaeris sessiliflora Extracts

The hydroethanolic dry extracts from flowers, leaves, and roots of *Hypochaeris sessiliflora* did not significantly reduce locomotion parameters or viability in *Caenorhabditis elegans* adults under the conditions tested. Therefore, no detectable nematode locomotion-disrupting activity was observed at the evaluated concentration and exposure times. Body-bend frequency and mean locomotion speed did not differ significantly from the vehicle control for any tissue extract (Figure 1A,B). The same conclusion was obtained for normalised speed and cumulative track distance (Figure S1A,B). Thus, at concentrations up to 1250 µg mL^−1^, these extracts showed no detectable locomotion-inhibitory activity.

This result defines a negative outcome for the extract type and assay used here. It should not be interpreted as evidence that *H. sessiliflora* lacks nematode-active metabolites. Hydroethanolic dry extraction favours polar constituents, including phenolic acids, glycosides, and saponins, whereas volatile or nonpolar compounds may be poorly represented. Nevertheless, other constituents not covered by the targeted method may also have been present and could have influenced the biological response. This distinction is relevant because nematode locomotion-disrupting activity reported for *Ageratum conyzoides*, another Asteraceae species, was associated with a hydrodistilled essential oil rich in precocene I and E-caryophyllene rather than with a polar hydroethanolic dry extract [32].

The absence of a significant locomotor response cannot be explained by the compositional data alone, as the individual compounds were not evaluated separately. Ferulic acid was the most abundant compound detected in all three plant parts. In *C. elegans*, ferulic acid has been studied mainly in disease models, where it reduced α-synuclein accumulation and improved dyskinesia through autophagy-related mechanisms [33]. These data do not predict nematode locomotion-disrupting activity in wild-type animals. Similarly, kaempferol and quercetin were identified in active fractions of *Annona crassiflora* that immobilised *C. elegans* larvae, but the activity of those fractions was not assigned to either compound alone [34]. Therefore, the presence of these phenolics in *H. sessiliflora* cannot be taken as evidence for or against nematode locomotion-disrupting activity.

Saponins were detected in all three plant parts, yet no locomotor effect was observed. This is not unexpected. Saponins are structurally diverse, and their biological effects in *C. elegans* are not uniformly toxic. Bitter melon saponins, for example, were reported to maintain or improve locomotor parameters rather than suppress them [35]. Since the saponins of *H. sessiliflora* were not structurally characterised, no mechanistic conclusion can be drawn from their qualitative detection alone.

The present assay detected strong locomotor inhibition, as shown by the levamisole control, but the study was not designed to detect weak effects or delayed toxicity. Under the conditions evaluated here, the conclusion is clear: hydroethanolic dry extracts of *H. sessiliflora* flowers, leaves, and roots did not impair adult *C. elegans* locomotion.

Although this study identified clear differences in the chemical and biological profiles of the flowers, leaves, and roots of *H. sessiliflora*, the detected compounds cannot be considered exclusive or species-specific markers. The targeted chromatographic approach was designed to quantify selected classes of known bioactive compounds rather than to discover previously undescribed metabolites. Similarly, the biological assays identified organ- and microorganism-dependent responses but did not demonstrate an activity unique to this species. The biological assays were performed using crude extracts, and the results therefore represent the net response of complex chemical mixtures. Therefore, untargeted metabolomic analyses, compound isolation and structural elucidation, comparisons with related *Hypochaeris* species, and bioactivity-guided fractionation will be required to identify potential chemotaxonomic markers or compounds responsible for the observed activities.

The present study evaluated locomotor responses in *C. elegans* under specific concentration and exposure conditions; therefore, it does not provide evidence of direct nematocidal activity. Further studies should assess mortality, survival over extended exposure periods, reproductive fitness, development, and offspring production to determine whether *H. sessiliflora* extracts exert nematicidal or sublethal effects.

## 4. Materials and Methods

### 4.1. Chemical Analysis of the Flowers, Leaves and Roots of Hypochaeris sessiliflora

**Figure 2:**
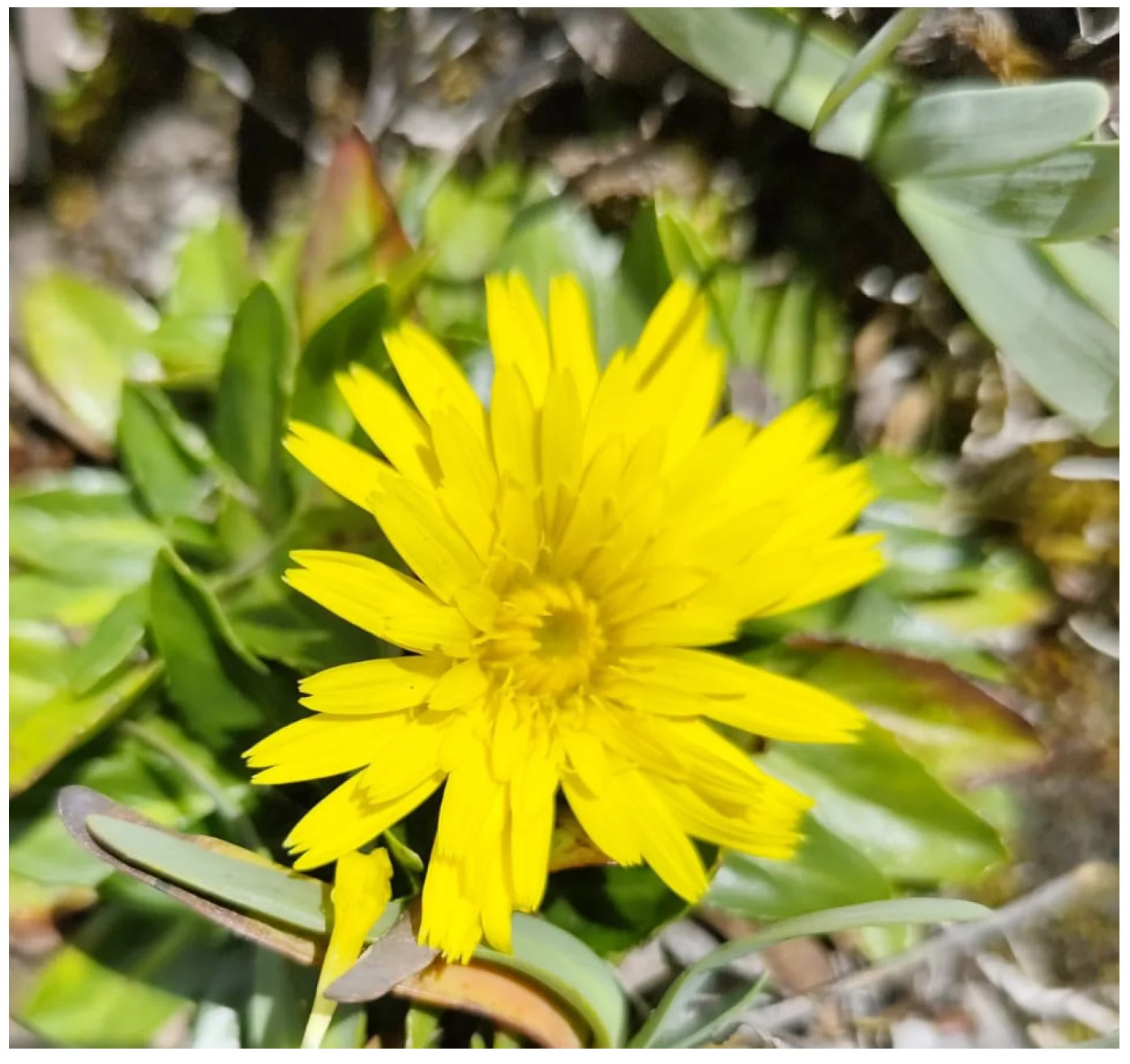
Photograph of ‘yellow chicory’ (*Hypochaeris sessiliflora)*.

The flowers, leaves, and roots of ‘yellow chicory’ (Figure 2) were collected in the Antisana moorland (0°30′40.2″ S, 78°12′54.8″ W) during March. The whole plant was pressed and botanically identified at the herbarium of the Universidad Politécnica Salesiana in Quito, Ecuador. Additionally, plant material was collected at random from the same area, comprising twenty plants. The flowers, leaves, and roots were collected separately to obtain two composite samples for each plant organ, which were divided into two portions. The first portion was used for physicochemical analysis, including determination of pH, soluble solids, titratable acidity, moisture, and ash content. The second portion was freeze-dried using a Christ Alpha 1-4 LDplus (GmbH, Osterode am Harz, Germany). Finally, the dried material was pulverised and stored until further analysis [36].

The preliminary phytochemical screening involved the qualitative detection of broad classes of secondary metabolites, including alkaloids, acetogenins, anthraquinones, steroids, terpenoids, phenols, flavonoids, tannins and saponins using group-specific chemical reactions. A preliminary qualitative phytochemical screening was performed to detect broad classes of secondary metabolites in aqueous extracts prepared from the freeze-dried flowers, leaves, and roots of *H. sessiliflora*. To this end, 20 mg of freeze-dried *H. sessiliflora* powder was mixed with 1 mL of deionised water. The suspension was homogenised and agitated for 3 min in an FS60D ultrasonic bath (Intebiolab Inc., Orlando, FL, USA). Subsequently, the supernatant was recovered by centrifugation at 14,000 rpm and 4 °C for 5 min in an Eppendorf 5430 centrifuge (Eppendorf AG, Hamburg, Germany). The solid residue was subjected to two further extractions, each using 500 µL of deionised water. Finally, the obtained supernatants were combined and used for phytochemical analysis, as described in the methodology of Coyago-Cruz et al. [37].

Calcium, iron, potassium and sodium were quantified by atomic absorption spectrophotometry using a Varian Spectra A-55 (Varian Inc., Palo Alto, CA, USA). The minerals were extracted in a Speed-180 wave Xpert microwave (Berghof Products + Instruments GmbH, Eningen unter Achalm, Germany) using Teflon digesters; for this, 40 mg of freeze-dried *H. sessiliflora* powder was mixed with 5 mL of 65% nitric acid and digested under a gradient according to the equipment specifications. The final digest was recovered and made up to 25 mL with deionised water. Calcium was measured at 422.7 nm, iron at 372.0 nm, potassium at 404.4 nm and sodium at 589.6 nm. Quantification was performed using calibration curves with R^2^ of 0.99, prepared from 1000 ppm standard solutions in deionised water, covering a concentration range of 0–200 ppm. The analysis was carried out in triplicate, and the mineral concentration was expressed in milligrams per 100 g of dry weight [36].

### 4.2. Bioactive Compounds by Chromatography

The assessment of bioactive compounds included quantification of vitamin C, organic acids, carotenoids, chlorophylls and their derivatives, and phenolic compounds. For each plant organ, three independent aliquots of the same homogenised composite sample were extracted separately. These were treated as analytical preparation replicates. All compounds were extracted from 20 mg of freeze-dried *H. sessiliflora* powder by microextraction, following homogenisation and agitation in an FS60 ultrasonic bath (Fisher Scientific Inc., Waltham, MA, USA). Subsequently, the supernatant was recovered by centrifugation in an Eppendorf 5430 (Eppendorf AG, Hamburg, Germany) at 14,000 rpm, 4 °C for 5 min. The extracts obtained were filtered through 0.45 µm PVDF filters. Chromatographic analysis was performed on an RRLC 1200 system (Agilent Technologies, Santa Clara, CA, USA) coupled to a DAD-UV-Vis detector, using wavelengths, chromatographic columns, injection volumes and mobile phases specific to each analysis. The mobile phase was maintained at a flow rate of 1 mL/min. Compound identification was carried out using the Lab ChemStation software (version 2.15.26), taking into account the characteristic spectra of each analyte and comparison with external analytical standards. Quantification was performed using calibration curves constructed with high-purity standards at a concentration of 1 mg/mL. All extractions were performed in triplicate, and each chromatographic run was performed in duplicate. Duplicate chromatographic injections were treated as technical replicates and averaged before statistical analysis. The independent sample preparations, rather than individual injections, were used as the experimental units.

The concentration reported for each chemical group was calculated as the sum of the individual compounds identified and quantified by the corresponding chromatographic method. Thus, total quantified organic acids, carotenoids, chlorophylls and their derivatives, and phenolic compounds represent summed analytical variables rather than measurements of the complete chemical classes present in the samples. Total quantified compounds were expressed as the sum of the individual compounds. Finally, the results were expressed as milligrams of the analyte per 100 g of dry weight [36,37,38].

Vitamin C was extracted using 1.2 mL of 3% metaphosphoric acid and 200 µL of 0.2% *DL*-homocysteine. The mixture was homogenised and then shaken for 1 min. Next, 600 µL of deionised water was added, followed by centrifugation and filtration of the supernatant. The resulting liquid extract was analysed on an RRLC 1200 chromatographic system at 244 nm, using a ZORBAX Eclipse XDB-C18 column (1.8 µm, 4.6 mm × 50 mm) (Agilent Technologies, Santa Clara, CA, USA). The mobile phase consisted of a solution of 1.5% monobasic potassium phosphate and 1.8% n-acetyl-n,n,n-trimethylammonium bromide dissolved in HPLC-grade methanol (90:10 *v*/*v*). The injection volume was 20 µL. Quantification was performed using *L*-(+)-ascorbic acid as a standard. Serial injections ranging from 1 to 20 µL were used to fit a linear regression model (R^2^ of 0.99). The method exhibited limits of detection (LODs) of 0.20 ppm and limits of quantification (LOQs) of 0.65 ppm. Quantification was performed using a calibration curve constructed with *L*-(+) ascorbic acid as the standard [36].

Organic acid was extracted using 1.5 mL of 0.02 N sulphuric acid with 0.05% metaphosphoric acid and 0.02% *DL*-homocysteine. The mixture was homogenised and then shaken for 3 min. Next, 500 µL of deionised water was added. The resulting liquid extract was analysed on an RRLC 1200 chromatographic system at 210 nm, using a YMC-Triart C18 column (3 µm, 4.6 mm × 150 mm) (YMC Europe GmbH, Dinslaken, Germany). The mobile phase consisted of a solution of 0.027% sulphuric acid. The injection volume was 20 µL. Quantification was performed using calibration curves for citric, malic, and L-(+)-tartaric acids prepared from 100 mg/mL stock solutions. Serial injections ranging from 1 to 20 µL were used to fit a linear regression model (R^2^ of 0.99). The method exhibited LODs and LOQs of 0.06 and 0.17 ppm for tartaric acid, 0.13 and 0.39 ppm for malic acid, and 0.08 and 0.26 ppm for citric acid, respectively. Total quantified organic acids were calculated as the sum of citric, malic, and tartaric acids [37].

Carotenoids, chlorophylls, and their derivatives were extracted using 1.0 mL of methanol:acetone:dichloromethane (1:1:2). The mixture was homogenised and then shaken for 1 min. The supernatant was collected, and the solid was re-extracted with 500 µL of dichloromethane as many times as necessary until the solid was colourless. The extract was evaporated to dryness in a Büchi R-100 rotary evaporator (Fisher Scientific, Hampton, NH, USA) at 30 °C. The dry extract was dissolved in ethyl acetate and quantified on an RRLC 1200 chromatographic system between 250 and 500 nm, using a YMC C30 column (3 µm, 4.6 × 150 mm) (YMC Europe GmbH, Dinslaken, Germany). The mobile phase consisted of methanol (A), methyl tert-butyl ether (B), and water (C), the gradient was 95% A + 5% B + 0% C at 0 min; 95% A + 5% B + 0% C at 5 min; 95% A+ 5% B + 0% C at 5 min; 89% A + 11% B + 10% C at 10 min; 89% A + 11% B + 0% C at 10 min; 75% A + 25% B + 0% C at 16 min; 40% A + 60% B + 0% C at 20 min; 15% A + 85% B + 0% C at 22 min; 90% A + 5% B + 5% C at 25 min; and 90% A + 5% B + 5% C at 28 min. The injection volume was 10 µL. Chromatographic peaks were identified using standards of α-carotene, β-carotene, β-cryptoxanthin, astaxanthin, trans-β-apo-8-carotenal, lycopene, lutein, violaxanthin, and zeaxanthin. A compound was considered identified when its retention time matched that of the corresponding standard within the predefined acceptance interval and its absorption spectrum matched the standard spectrum. The major compounds were quantified using individual calibration curves at 1 mg/mL. Serial injections ranging from 1 to 20 µL were used to fit a linear regression model (R^2^ of 0.99). Total quantified carotenoids were calculated as the sum of individual compounds. The method exhibited LODs and LOQs of 0.00 and 0.02 ppm for lutein, 0.029 and 0.09 ppm for β-carotene, and 0.003 and 0.008 ppm for zeaxanthin, respectively.

On the other hand, quantification of chlorophyll and derivatives was carried out using calibration curves for pheophytin a, pheophytin b, chlorophyll a, and chlorophyll b. Total chlorophyll and its derivatives were calculated as the sum of individual compounds [38].

Phenolics were extracted using 1.0 mL of 80% methanol acidified with 0.1% HCl. The mixture was homogenised and then shaken for 3 min. The supernatant was collected, and the solid was twice re-extracted with 500 µL of acidified methanol. The resulting liquid extract was analysed on an RRLC 1200 chromatographic system at 200–500 nm, using a Zorbax Eclipse Plus C18 column (4.6 × 150 mm, 5 µm) (Agilent Technologies, Santa Clara, CA, USA). The mobile phase consisted of 0.01% aqueous formic acid (A) and acetonitrile (B); the gradient was 0 min, 100% A; 5 min, 95% A + 5% B; 20 min, 50% A + 50% B; 30 min, column wash and re-equilibration. The injection volume was 10 µL. Chromatographic peaks were identified comparing the retention time and DAD-UV-Vis absorption spectrum of each sample peak with those obtained from an authentic analytical standards of *p*-hydroxybenzoic acid, 3-hydroxybenzoic acid, 2-methoxybenzoic acid, 3-methoxybenzoic acid, 2,5-dihydroxybenzoic acid, gallic acid, protocatechuic acid, vanillic acid, shikimic acid, syringic acid, ellagic acid, tannic acid, *p-*, *m*- and *o*-coumaric acids, as well as chlorogenic, caffeic and ferulic acids, rutin, quercetin, myricetin, kaempferol, quercetin glucoside, chrysin, luteolin, catechin, naringenin and naringin were used. The major compounds were quantified using individual calibration curves at 1 mg/mL. Serial injections ranging from 1 to 20 µL were used to fit a linear regression model (R^2^ of 0.99). Total quantified phenolics were calculated as the sum of individual compounds [36]. The method exhibited LODs and LOQs of 0.009 ppm and 0.028 ppm for chlorogenic acid; 0.048 ppm and 0.145 ppm for caffeic acid; 0.012 ppm and 0.041 ppm for ferulic acid; 0.006 ppm and 0.014 ppm for *p*-hydroxybenzoic acid; and 0.007 ppm and 0.021 ppm for gallic acid, respectively.

### 4.3. In Vitro Chemical Antioxidant Activity by Spectrophotometry

In vitro chemical antioxidant activity was assessed using the ABTS and DPPH methods with a BioTek H1 spectrophotometer fitted with a microplate reader (Agilent Scientific Instruments, Santa Clara, CA, USA). The ABTS and DPPH assays were performed to compare the radical-scavenging capacity of the flowers, leaves, and roots of *H. sessiliflora*. In vitro chemical antioxidant activity was determined using three independently prepared extracts from the same homogenised composite sample. The extract was prepared by mixing 20 mg of freeze-dried *H. sessiliflora* powder with 2 mL of methanol, homogenising and agitating for 3 min in an FS60 ultrasonic bath (Fisher Scientific Inc., Waltham, MA, USA). Subsequently, the supernatant was recovered by centrifugation in an Eppendorf 5430 (Eppendorf AG, Hamburg, Germany) at 14,000 rpm, 4 °C for 5 min. The extracts obtained were filtered through 0.45 µm PVDF filters. All extractions were performed in triplicate, and each reading was performed in duplicate. Quantification was performed using a calibration curve with Trolox standard at a concentration of 0.2 mg/mL. Each extract was measured in four wells, treated as technical replicates, and averaged before statistical analysis. The results were expressed as millimoles of Trolox equivalents per 100 g of dry weight [37].

The ABTS radical was prepared by mixing equal volumes of a 7 mM ABTS solution with a 2.45 mM potassium persulfate solution. The mixture was left to stand in the dark for 16 h and subsequently diluted with methanol until a final absorbance of 0.7 at 734 nm was achieved. To quantify in vitro chemical antioxidant activity, 10 µL of the extract was mixed with 200 µL of the ABTS radical solution. The mixture was homogenised, and the absorbance was recorded on the spectrophotometer after a 15 min reaction.

The DPPH radical was prepared by dissolving 10 mg of DPPH in 50 mL of methanol. The solution was adjusted to an absorbance of 0.9–1.1 at 515 nm. For quantification, 20 µL of the extract was mixed with 180 µL of the DPPH solution. The mixture was then incubated for 40 min at room temperature in the dark before spectrophotometric measurement.

### 4.4. Antimicrobial Activity

Crude extracts were used in the biological assays because the study was designed as an initial screening of the overall extractable chemical fraction of each plant organ rather than as an evaluation of isolated compounds. The crude hydroethanolic dry extracts were obtained using an extraction procedure distinct from the compound-specific procedures used for the compositional analysis of the dried plant material. Thus, the hydroethanolic dry extract of flowers, leaves and roots of *H. sessiliflora* was prepared by mixing 2 g of freeze-dried powder with 25 mL of 50% ethanol, homogenising, and agitating for 6 min. The supernatant was collected, and the solid was twice re-extracted with 25 mL of ethanolic solution. The hydroethanolic dry extract was filtered through Whatman No. 1 filter paper, then concentrated on a rotary evaporator and freeze-dried. Extraction yield (%) was calculated as the mass of the freeze-dried hydroethanolic extract divided by the initial mass of freeze-dried plant material and multiplied by 100.

Total phenolic content was determined using the Folin–Ciocalteu microplate method described by Coyago-Cruz et al. [39]. The extracts considered for the study were the phenolic extract described in Section 2.4. For the hydroethanolic dry extracts, approximately 20 mg of each extract was reconstituted in 2 mL of water. The mixture was homogenised using a vortex mixer, sonicated for 3 min, and centrifuged at 14,000 rpm for 5 min at 4 °C. The resulting supernatant was collected and filtered through a 0.45 µm PVDF filter.

In a 96-well microplate, 20 µL of the filtered sample extract was mixed with 100 µL of Folin–Ciocalteu reagent diluted 1:4 (*v/v*) with deionised water. Subsequently, 75 µL of sodium carbonate solution (100 g/L) was added, and the microplate was shaken for one min. After incubation for 2 h at room temperature in the dark, absorbance was measured at 750 nm using a BioTek Synergy H1 microplate reader (Agilent Technologies, Santa Clara, CA, USA). Quantification was performed using a gallic acid calibration curve ranging from 10 to 200 mg/L, with an R^2^ of 0.99. Three independently prepared analytical replicates were analysed for each plant organ, and each measurement was performed quadruplicate. Results for the freeze-dried plant material were expressed as mg of gallic acid equivalents per 100 g of dry plant material (mg GAE/100 g DW), whereas results for the hydroethanolic extracts were expressed as mg of gallic acid equivalents per gram of dry extract (mg GAE/g DE).

#### 4.4.1. ATCC Microorganisms

The antibacterial assay against reference ATCC strains was conducted as an initial screening to determine the inhibitory spectrum of the crude extracts. The antibacterial and antifungal activities of the hydroethanolic dry extract of flowers, leaves and roots of *H. sessiliflora* were evaluated using the broth microdilution method according to CLSI guidelines, employing the bacterial strains *Escherichia coli* ATCC 8739, *Pseudomonas aeruginosa* ATCC 9027, *Staphylococcus aureus* ATCC 6538P, *Streptococcus mutans* ATCC 25175, *Pseudomonas fluorescens* ATCC 13525, *Staphylococcus epidermidis* ATCC 12228, *Pseudomonas putida* ATCC 31483, *Edwardsiella tarda* ATCC 15947, *Enterococcus faecalis* ATCC 49533, *Klebsiella pneumoniae* ATCC 10031 and *Listeria monocytogenes* ATCC 19114. For bacteria, a stock solution was prepared by dissolving 400 mg of lyophilised extract in 2 mL of sterile distilled water, followed by twofold serial dilution in sterile 96-well microplates containing 100 µL of BHI broth per well; 100 µL of the stock solution was added to the first column and subsequently transferred across the columns. Each well was inoculated with 20 µL of a standardised microbial suspension (OD600 = 0.2; ~1.5 × 10^8^ CFU/mL), adjusted to yield approximately 5 × 10^5^ CFU per well, in a final volume of 120 µL. Microplates were incubated at 37 °C for 24 h for *E. coli*, *S. aureus*, *P. aeruginosa*, *S. mutans*, *P. putida*, *S. epidermidis*, *E. faecalis*, *K. pneumoniae*, *L. monocytogenes*, *and E. tarda*, while *P. fluorescens* was incubated at 29 °C for the same duration.

After incubation, cell viability was assessed by adding 20 µL of TTC to each well and reincubating for 1–2 h in the dark, evaluating formazan formation as an indicator of metabolic activity to determine the MIC. For yeast, results were confirmed by a spot test on SDA plates, depositing 4 µL from each well and assessing growth after an additional 24 h of incubation, for a total of 48 h. All assays were performed in triplicate to ensure reproducibility. Streptomycin and fluconazole were used as positive reference controls for the bacterial and fungal strains, respectively. Growth and sterility controls were included, and the solvent was evaluated at the same final concentration used for the extract treatments. These reference controls were processed under the same incubation conditions as the experimental samples. The assay followed broth microdilution principles adapted from CLSI recommendations for testing crude plant extracts; therefore, the measured MICs represent experimental inhibitory concentrations and were not interpreted as clinical susceptibility breakpoints.

#### 4.4.2. Antifungal Activity

The antifungal assay was performed to determine whether the crude extracts inhibited the growth of four clinically relevant *Candida* species under the evaluated concentration range. The antifungal potential of hydroethanolic dry extracts prepared from *H. sessiliflora* root, flowers and leaves was assessed against four *Candida* species, namely *C. albicans*, *C. glabrata*, *C. krusei*, and *C. tropicalis*, by means of a modified broth microdilution assay coupled to growth-kinetics monitoring [5]. Briefly, each strain was cultured overnight in YPD broth, after which the cell suspension was adjusted with sterile distilled water to an OD600 of 0.1. The standardised cultures were subsequently diluted in YPD medium to prepare the inoculum for the assay.

The experiments were carried out in sterile 96-well microplates using YPD medium, with a final volume of 200 µL per well. Flower, root, and leaf hydroethanolic dry extracts were evaluated independently at final concentrations of 1250, 625, 312.5, 156.25, and 78.125 µg mL^−1^. Extracts were dissolved in DMSO, and the assay was designed so that all extract-treated wells contained 2.5% DMSO as the final vehicle concentration. Consequently, throughout the concentration series, the extract dose varied while the DMSO concentration remained constant. Growth controls, vehicle controls, and medium-only blanks were included in each microplate.

Plates were incubated at 35 °C in a Cytation 5 multimode reader, and fungal growth was followed for 48 h by recording OD600 values every 30 min. Antifungal activity was evaluated by comparing the growth curves obtained for each extract treatment with those of the corresponding controls. Each condition was tested in three technical replicates, and the complete experiment was performed in three independent assays.

#### 4.4.3. Multidrug-Resistant Microorganisms

Assays against multidrug-resistant clinical isolates were conducted to explore whether the crude extracts exhibited detectable growth-inhibitory activity against bacterial strains with clinically relevant resistance profiles. The antibacterial activity of the hydroethanolic dry extract of the flowers, leaves and roots of *H. sessiliflora* was assessed against seven multidrug-resistant bacteria: *Klebsiella pneumoniae*, *Escherichia coli*, *Salmonella enterica serovar Kentucky*, *Enterococcus faecalis*, *Staphylococcus epidermidis*, *Enterococcus faecium*, and *Pseudomonas aeruginosa* using the microdilution method. These seven clinical multidrug-resistant isolates were provided by the National Health Institute of Ecuador (INSPI).

The bacterial inoculum was prepared in brain–heart infusion broth (BHI) to a final cell density of 5 × 10^5^ CFU/mL. Stock solutions of the tested extracts were prepared by dissolving them in DMSO at 25 mg/mL for flowers and leaves and at 80 mg/mL for roots. Tested volumes were adjusted so the final concentration of DMSO in each well was always 2.5% (*v*/*v*). This concentration was previously shown not to affect bacterial growth [40]. As a control, bacterial cells were grown in the presence of 2.5% DMSO to rule out any potential growth-inhibitory effect. Additionally, Nourseothricine (100 µg/mL) was used as a control for growth inhibition. Both BHI alone and supplemented with the extract were used as blanks.

Extract sensitivities were assessed using the microdilution method according to the Clinical and Laboratory Standards Institute (CLSI) guidelines [41], with the following modifications: first, 5 µL of each extract was added to 195 µL of bacterial suspension (5 × 10^5^ CFU/mL) to a final volume of 200 µL. The final concentrations at the wells were 125 µg/mL for flowers and leaves, and 2 mg/mL for roots. The plates were then incubated at 37 °C for 20 h with constant shaking at 300 cpm (double orbital setting), and the OD_600_ was monitored every 30 min using a Cytation 5 multi-mode detection system (BioTek Instruments, Winooski, VT, USA). The minimum inhibitory concentration (MIC) was determined by comparing the OD600 at 0 h with that at 24 h for samples exposed to the test suspension. These assays were performed at least in triplicate.

### 4.5. Screening of Nematode and Locomotion-Disrupting Activities in Caenorhabditis elegans

The *C. elegans* assay was performed to determine whether the crude ethanolic extracts exhibited detectable effects on nematode locomotion. Activity of hydroethanolic dry extracts prepared from flowers, leaves, and roots of *Hypochaeris sessiliflora* was assessed using synchronised L1 larvae of the wild-type *Caenorhabditis elegans* strain N2. Larval synchronisation was achieved by hypochlorite treatment of gravid hermaphrodites, followed by overnight hatching in sterile M9 buffer at 20 °C. To ensure developmental homogeneity, only freshly hatched L1 larvae collected within 12–16 h after hatching were used in the assays.

*Escherichia coli* OP50 was used as the bacterial food source. OP50 cultures were grown overnight in LB medium at 37 °C with shaking, harvested by centrifugation, and inactivated by UV exposure for 30 min. The UV-killed bacteria were washed three times with sterile M9 buffer by centrifugation at 4400 rpm for 5 min per wash to remove residual culture medium and soluble metabolites. After the final wash, the bacterial pellet was resuspended in sterile M9 buffer and adjusted to an OD600 of 0.4.

For each experiment, a master suspension was prepared in sterile M9 buffer containing synchronised L1 larvae, UV-inactivated OP50, and cholesterol. The number of larvae was adjusted to dispense approximately 50 L1 larvae per well. OP50 was incorporated into the master suspension at an OD600 of 0.4, yielding an approximate final OD600 of 0.2 in the assay wells, whereas cholesterol was included at a final concentration of 5 µg mL^−1^. The suspension was gently homogenised immediately before dispensing to maintain an even distribution of larvae and bacteria.

Hydroethanolic dry extracts from flowers, leaves, and roots were dissolved in DMSO to prepare stock solutions and were evaluated independently. The assays were performed in sterile flat-bottom 96-well microplates in a final volume of 150 µL per well. Each well first received 75 µL of the corresponding extract dilution or control solution, followed by 75 µL of the larval master suspension. Wells containing only the vehicle were used as negative controls, whereas levamisole hydrochloride at a final concentration of 50 mM was included as a positive control for paralysis and mortality. Solutions were filter-sterilised when necessary.

To limit evaporative loss during incubation, sterile M9 buffer was added to the interwell spaces and peripheral reservoirs of the plates. Microplates were incubated at 21 °C for up to 72 h under continuous gentle orbital agitation at 110 rpm, thereby maintaining larvae in suspension and promoting uniform exposure to the extracts.

Larval motility and viability were evaluated at the start of the assay and subsequently every 24 h for 72 h. Motility was determined from video recordings and quantified using the wrMTrck plugin in Fiji/ImageJ (ImageJ 1.54p). Each treatment was evaluated with at least 8 technical replicates, and the entire experiment was independently repeated at least 3 times.

Extracts were considered to exhibit nematode locomotion-disrupting activity when they produced a statistically significant decrease in motility and/or viability compared with the vehicle control (*p* < 0.05).

### 4.6. Statistical Analysis

Results are expressed as the mean ± SD or SEM, as specified. For each physicochemical, mineral, bioactive-compound, and in vitro chemical antioxidant variable, differences among flowers, leaves, and roots were evaluated using a one-way analysis of variance (ANOVA), with plant organ as the fixed factor. The experimental unit was an independently prepared sample extract, and three independent preparations were analysed per plant organ (n = 3). Normality of the residuals and homogeneity of variances were assessed using the Shapiro–Wilk and Levene tests, respectively. When the ANOVA indicated a significant effect, pairwise comparisons were performed using Tukey’s honestly significant difference test, with family-wise error controlled at α = 0.05.

Locomotion parameters were obtained from individual *C. elegans* tracks generated with WrmTrck/Fiji. For each video, all track values were summarised as a single median, which was used as the experimental unit for statistical analysis. Thus, each condition included three independent observations (n = 3 videos).

Differences among conditions were evaluated for body-bend frequency (BBPS, bends s^−1^), mean locomotion speed (AvgSpeed, µm s^−1^), normalised speed (BLPS, body lengths s^−1^), and cumulative track distance (µm). Global comparisons were performed using the Kruskal–Wallis rank test. Pairwise comparisons between the vehicle control (C−) and each extract were performed using two-sided Mann–Whitney U tests.

Effect size was estimated as the rank-biserial correlation coefficient, calculated as r = 1 − 2U/(n_1_ × n_2_), where U is the Mann–Whitney statistic and n_1_ and n_2_ are the group sizes. Because n_1_ = n_2_ = 3, the smallest attainable two-sided Mann–Whitney *p*-value is 0.100; therefore, Bonferroni correction was not applied. Uncorrected *p*-values are reported together with effect sizes. A trend was considered biologically relevant when *p* ≤ 0.100 and r = −1.000, indicating complete rank separation between groups. All analyses were performed in Python 3.12 using SciPy v1.17.

## 5. Conclusions

The Andean moors are largely unexplored regions home to a wealth of flora and fauna. This exploratory study therefore demonstrated that different parts of *Hypochaeris sessiliflora* exhibited clearly distinct phytochemical and bioactive profiles. The flowers contained the highest summed concentrations of quantified phenolic compounds and carotenoids and also exhibited the greatest antioxidant activity according to the ABTS assay. However, because the activity was measured in complex extracts, the contribution of individual compounds to this response could not be established. The leaves stood out for their high content of vitamin C, organic acids, minerals and chlorophylls. On the other hand, the roots exhibited a more limited chemical profile, although they displayed the highest antimicrobial activity against *Staphylococcus epidermidis*. The extracts showed an antimicrobial spectrum that depended on both the plant organ and the microorganism tested, with no activity observed against *Candida* species or against multidrug-resistant bacterial strains under the experimental conditions used. The hydroethanolic dry extracts obtained from the flowers, leaves, and roots of *H. sessiliflora* did not exhibit detectable antibacterial activity against the panel of multidrug-resistant clinical isolates. Furthermore, the absence of significant alterations in *Caenorhabditis elegans* locomotion suggests that the hydroethanolic dry extracts do not produce detectable neuromotor effects in the biological model used under the concentration and exposure conditions evaluated. Overall, this study establishes an initial organ-specific chemical and functional profile of *H. sessiliflora*. Although no compound or biological activity can currently be considered unique to this species, the predominance of phenolic compounds and carotenoids in the flowers, together with their antioxidant activity and the selective antimicrobial responses of the extracts, allows for the identification of specific plant organs and chemical fractions that warrant further investigation.

## Figures and Tables

**Table 1:** Preliminary qualitative phytochemical screening of the aqueous extracts prepared from the flowers, leaves, and roots of *H. sessiliflora*.

Metabolites	Flowers	Leaves	Roots
Alkaloids	−	−	−
Acetogenins	−	−	−
Anthraquinones	−	−	−
Steroids	+	−	−
Terpenoids	−	−	−
Phenols	+	−	+
Flavonoids	−	−	−
Tannins	+	−	+
Saponins	+	+	+

Note: +, indicates a presumptive positive response in the corresponding group-specific qualitative assay; −, indicates that no characteristic response was observed under the screening conditions used.

**Table 2:** Average values from chemical analysis and mineral concentrations in the plant parts of *H. sessiliflora*.

Parameters	Flowers	Leaves	Roots
pH	5.9 ± 0.0 ^a^	5.8 ± 0.0 ^a^	5.7 ± 0.0 ^a^
SS (°Brix)	1.0 ± 0.0 ^b^	0.1 ± 0.0 ^c^	2.7 ± 0.3 ^a^
TA (%)	0.1 ± 0.0 ^a^	0.1 ± 0.0 ^a^	0.1 ± 0.0 ^a^
Humidity (%)	83.0 ± 2.3 ^a^	82.8 ± 0.8 ^a^	69.3 ± 2.9 ^b^
Ash (%)	1.2 ± 0.1 ^b^	2.3 ± 0.2 ^a^	0.7 ± 0.1 ^c^
Mineral profile (mg/100 g DW)			
Ca	593.0 ± 2.0 ^b^	1163.1 ± 28.0 ^a^	312.8 ± 0.0 ^c^
Fe	20.6 ± 0.1	nd	nd
K	2372.1 ± 0.0 ^b^	3256.6 ± 0.1 ^a^	1072.4 ± 0.1 ^c^
Na	54.4 ± 3.0 ^a^	47.3 ± 16.4 ^b^	47.6 ± 2.6 ^b^

Note: mean ± standard deviation (n = 3). Lowercase letters followed by the standard deviation indicate differences in means among flowers, leaves, and roots, with *p* < 0.05. SS, soluble solids; TA, titratable acidity; nd, undetectable.

**Table 3:** Concentrations of bioactive compounds quantified in the dried flowers, leaves, and roots of *H. sessiliflora*.

Parameters	Flowers	Leaves	Roots
Vitamin C (mg/100 g DW)	35.5 ± 2.9 ^a^	39.2 ± 2.9 ^a^	18.0 ± 0.1 ^b^
Organic acid (mg/100 g DW)			
Citric acid	146.9 ± 2.0 ^c^	252.0 ± 11.2 ^a^	164.0 ± 1.4 ^b^
Malic acid	219.6 ± 19.6 ^b^	463.1 ± 11.9 ^a^	68.8 ± 1.1 ^c^
Tartaric acid	287.4 ± 81.6 ^b^	498.4 ± 8.5 ^a^	164.4 ± 7.6 ^c^
Total quantified organic acid	653.9 ± 16.1 ^b^	1213.5 ± 31.5 ^a^	397.2 ± 7.3 ^c^
Carotenoids (mg/100 g DW)			
α-Carotene	67.0 ± 5.5	-	-
Lutein	8.8 ± 0.3 ^b^	15.6 ± 1.0 ^a^	0.1 ± 0.0 ^c^
9-*Cis*-anteraxanthin	1.0 ± 0.0 ^a^	1.0 ± 0.0 ^a^	-
Zeaxanthin	0.5 ± 0.1 ^a^	0.7 ± 0.1 ^a^	-
β-Carotene	74.8 ± 0.0	-	-
Total quantified carotenoid	152.1 ± 5.7 ^a^	17.3 ± 2.4 ^b^	0.1 ± 0.0 ^c^
Chlorophylls and their derivatives (mg/100 g DW)			
Chlorophyll b	14.8 ± 0.8 ^b^	45.0 ± 3.5 ^a^	-
Pheophytin a	-	44.0 ± 3.7	-
Total quantified chlorophyll	14.8 ± 0.8 ^b^	89.0 ± 7.2 ^a^	-
Phenolic compounds (mg/100 g DW)			
Gallic acid	2.0 ± 0.1 ^b^	2.8 ± 0.7 ^a^	2.3 ± 0.1 ^ab^
4-Hydroxibenzoic acid	17.0 ± 0.7 ^a^	6.7 ± 0.4 ^b^	-
*m*-Coumaric acid	-	-	531.6 ± 31.2
Syringic acid	23.7 ± 0.1	-	-
Ferulic acid	11,902.7 ± 90.9 ^a^	5794.6 ± 43.9 ^b^	845.9 ± 19.2 ^c^
Rutin	223.6 ± 1.0 ^b^	435.7 ± 24.2 ^a^	-
Kaempferol	172.1 ± 9.6 ^a^	32.2 ± 0.8 ^b^	-
Total quantified phenolics	12,341.1 ± 91.4 ^a^	6272.0 ± 46.5 ^b^	1379.8 ± 50.5 ^c^

Note: Values are expressed as mean ± standard deviation (n = 3). Lowercase letters followed by the standard deviation indicate differences in means among flowers, leaves, and roots, with *p* < 0.05. Values are expressed on a dry-weight basis and correspond to compounds quantified directly in the dried plant material using compound-specific extraction and chromatographic procedures. -, No presence was detected under the conditions analysed.

**Table 4:** Average in vitro chemical antioxidant activity values in the plant parts of *H. sessiliflora*.

Parameters	Flowers	Leaves	Roots
DPPH (mmol TE/100 g DW)	2.8 ± 0.2 ^a^	2.9 ± 0.1 ^a^	2.9 ± 0.0 ^a^
ABTS (mmol TE/100 g DW)	6.1 ± 0.0 ^a^	5.8 ± 0.0 ^b^	1.5 ± 0.1 ^c^

Note: Values are expressed as mean ± standard deviation (n = 3). Lowercase letters followed by the standard deviation indicate differences in means among flowers, leaves, and roots, with *p* < 0.05.

**Table 5:** Total phenolic content of the freeze-dried plant material and corresponding hydroethanolic dry extracts of *H. sessiliflora*.

Matrix	Unit	Flowers	Leaves	Roots
Freeze-dried plant material	mg GAE/100 g DW	16,673.7 ± 85.3 ^b^	6482.2 ± 98.3 ^a^	530.2 ± 2.6 ^c^
Hydro-ethanolic dry extracts	mg GAE/100 g DE	23,895.3 ± 97.9 ^a^	21,563.2 ± 71.1 ^b^	5934.2 ± 17.4 ^c^
Phenolics recovered relative to plant material	mg GAE/100 g DW	2652.4	7008.0	421.3

Note: Values are expressed as mean ± standard deviation (n = 3). Lowercase letters followed by the standard deviation indicate differences in means among flowers, leaves, and roots, with *p* < 0.05. DW, dry weight of plant material; DE, dry extract; GAE, gallic acid equivalents. Phenolics recovered relative to plant material were calculated using the total phenolic content of the dry extract and the corresponding extraction yield.

**Table 6:** Minimum inhibitory concentration (MIC) in the plant parts of *H. sessiliflora*.

Bacterial Strain	MIC (mg/mL)		
	Flowers	Leaves	Roots
*E. coli* ATCC 8739	31.2	62.6	62.5
*P. aeruginosa* ATCC 9027	-	125.1	-
*S. aureus* ATCC 6538P	31.2	15.6	125.0
*S. mutans* ATCC 25175	62.5	-	62.5
*P. fluorescens* ATCC 13525	-	-	-
*S. epidermidis* ATCC 12228	-	116.2	8.2
*P. putida* ATCC 31483	-	-	-
*E. faecalis* ATCC 49533	-	162.5	150.1
*K. pneumoniae* ATCC 10031	-	162.5	150.1
*L. monocytogenes* ATCC 19114	154.1	81.3	75.0
*E. tarda* ATCC 15947	12.8	14.5	130.6

Note: - indicates no activity at the tested concentrations. The standard deviation for these values is zero in all cases.

## Data Availability

The original contributions presented in this study are included in the article. Further inquiries can be directed to the corresponding author.

## Supplementary Materials

The following supporting information can be downloaded at: https://www.mdpi.com/article/10.3390/ph19081314/s1. Figure S1: Additional locomotion parameters of *Caenorhabditis elegans* treated with *Hypochaeris sessiliflora* ethanolic extracts.
