# Supplementary material for: Unexplored Bioactive, Antioxidant, Antimicrobial, and Nematicidal Potential of the Flowers, Leaves, and Roots of ‘Yellow Chicory’ (Hypochaeris sessiliflora)
Source: Pharmaceuticals (Basel). 2026 Aug 20;19(8):1314. doi: 10.3390/ph19081314 (PMC13516099; doi:10.3390/ph19081314)
Supplement: Supplementary file 1 [file pharmaceuticals-19-01314-s001.zip › pharmaceuticals-4437509-supplementary.pdf]

Unexplored Bioactive, Antioxidant, Antimicrobial, and Nematicidal Potential of the Flowers, Leaves, and Roots of 'Yellow Chicory' (*Hypochaeris sessiliflora*)

Elena Coyago-Cruz [1,*], Gabriela Méndez [1], Johana Zúñiga-Miranda [2], Alejandro Porras [3], Carlos Barba-Ostria [4,5], Ivanna Sarabia [1], Linda P. Guamán [2], Celina Coello [1], Jorge Heredia-Moya [2], Blanca Naranjo [3] and María Claudia Segovia-Salcedo [3]

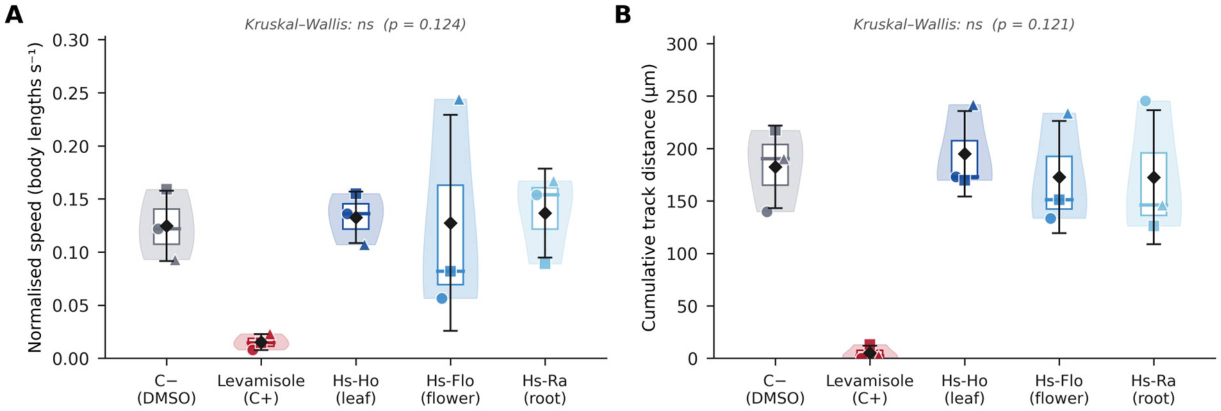

Supplementary Figure S1. Additional locomotion parameters of Caenorhabditis elegans treated with Hypochaeris sessiliflora ethanolic extracts. Normalized locomotion speed (A) and cumulative track distance (B) were measured in wild-type C. elegans N2 animals treated with H. sessiliflora leaf (Hs-Ho, 500 µg mL^{-1}), flower (Hs-Flo, 125 µg mL^{-1}), or root (Hs-Ra, 500 µg mL^{-1}) ethanolic extracts. Vehicle-treated animals were used as negative control (C−; 2.5% DMSO), and levamisole as positive control (C+; 20 µg mL^{-1}). Data were acquired and processed as described for Figure 2. Each condition included three independent 20-s videos; each point represents the median value of all tracks detected in one video. Circles, squares, and triangles indicate Videos 1, 2, and 3, respectively. Violin plots show the distribution of per-video medians; white boxes indicate the interquartile range, horizontal lines the median, black diamonds the mean, and error bars ±1 SD. Colors indicate conditions: grey, C−; red, C+; dark blue, Hs-Ho; medium blue, Hs-Flo; light blue, Hs-Ra. No significant pairwise differences were detected between any H. sessiliflora extract and C− for either parameter. Kruskal–Wallis H and p values are shown above each panel. Measurements were calibrated using 280 pixels = 1,000 µm. BLPS is expressed as body lengths s^{-1}. Cumulative track distance is expressed in µm and corresponds to the total path length recorded for each worm track.
